# Crystal structure of the 1,3,6,8-tetra­aza­tri­cyclo[4.3.1.1^3,8^]undecane (TATU)–4-nitro­phenol (1/2) adduct: the role of anomeric effect in the formation of a second hydrogen-bond inter­action

**DOI:** 10.1107/S2056989015019659

**Published:** 2015-10-24

**Authors:** Augusto Rivera, Héctor Jairo Osorio, Juan Manuel Uribe, Jaime Ríos-Motta, Michael Bolte

**Affiliations:** a Universidad Nacional de Colombia, Sede Bogotá, Facultad de Ciencias, Departamento de Química, Cra 30 No. 45-03, Bogotá, Código Postal 111321, Colombia; b Universidad Nacional de Colombia, Sede Manizales, Colombia; c Institut für Anorganische Chemie, J. W. Goethe-Universität Frankfurt, Max-von Laue-Stasse, 7, 60438 Frankfurt/Main, Germany

**Keywords:** crystal structure, cocrystalline adducts, hydrogen bonding

## Abstract

In the title ternary co-crystalline adduct, C_7_H_14_N_4_·2C_6_H_5_NO_3_, mol­ecules are linked by two inter­molecular O—H⋯N hydrogen bonds, forming a tricomponent aggregates in the asymmetric unit. The hydrogen-bond formation to one of the N atoms is enough to induce structural stereoelectronic effects in the normal donor→acceptor direction. In the title adduct, the two independent nitro­phenol mol­ecules are essentially planar, with maximum deviations of 0.0157 (13) and 0.0039 (13) Å. The dihedral angles between the planes of the nitro group and the attached benzene rings are 4.04 (17) and 5.79 (17)°. In the crystal, aggregates are connected by C—H⋯O hydrogen bonds, forming a supra­molecular dimer enclosing an *R*_6_^6^(32) ring motif. Additional C—H⋯O inter­molecular hydrogen-bonding inter­actions form a second supra­molecular inversion dimer with an *R*_2_^2^(10) motif. These units are linked *via* C—H⋯O and C—H⋯N hydrogen bonds, forming a three-dimensional network.

## Abstract 2

The components of the ternary cocrystalline adduct are linked by inter­molecular O—H⋯N hydrogen bonds This is the first example of a 1:2 adduct containing two 4-nitro­phenol mol­ecules and one aza­adamantane structure.

## Chemical context   

The chemistry of the amino­alkyl­ation of aromatic substrates by the Mannich reaction is of great inter­est and chemical importance (Tramontini *et al.*, 1988 ▸). Some modern variants of Mannich reactions have been developed using preformed aminals or hemiaminals as Mannich electrophiles for amino­methyl­ation reactions (Katritzky *et al.*, 2005 ▸). The use of these preformed amino­methyl­ating reagents, particularly those derived from common amines, is becoming more frequent (Tramontini & Angiolini, 1990 ▸), a mechanism involving initial formation of a hydrogen-bonded complex between a Mannich preformed reagent and the phenolic substrate has been documented (Burckhalter & Leib, 1961 ▸). Electron density at the free *ortho* position of the phenol and the reactivity of the phenolic hy­droxy group played a crucial role and the reaction does not occur in the absence of the hy­droxy group (Deng *et al.* 2014 ▸). The phenol–N complex has also served as a good model for the investigation of proton and electron-transfer processes occurring in living matter, it being generally assumed that this inter­action consists solely of the attraction between the lone pair of the amine N atom and the phenolic hy­droxy proton (Lu *et al.* 2006 ▸). In addition to the typical features of inter­molecular hydrogen bonding, these systems have an extra advantage over many other complexes because they play an important role in probing the anomeric effect in N—C—N (aminal) systems even though the anomeric effect is well recognized as an important factor in defining the predominant conformational state of many cyclic heteroatom-containing compounds (Dabbagh *et al.* 2002 ▸). There is little evidence in the literature for bond shortening and lengthening in cyclic aminals (Takahashi *et al.* 2007 ▸). We have undertaken a long-term project designed to systematically investigate the structures, chemical properties and reactivity of macrocyclic aminals as preformed electrophilic reagents for the synthesis of phenolic Mannich bases through simple and efficient methodologies. As part of this investigation we have recently reported the synthesis of 1,3,6,8-tetra­aza­tri­cyclo­[4.3.1.1^3,8^]undecane (TATU), (II) (Rivera *et al.* 2004 ▸). TATU which has two non-equivalent hydrogen-bond acceptor N-atom sites is a good model for the investigation of inter­molecular hydrogen bonding with phenols and for studying the nature of the anomeric effect in the N—C—N mol­ecular segment. One inter­esting feature of the structure of TATU is that two of the N atoms are similar to those in 1,3,5,7-tetra­aza­tri­cyclo­[3.3.1.1^3,7^]decane [(III), also known as urotropine, and hexa­methyl­ene­tetra­mine, HMTA] and the other two are similar to those in 1,3,6,8-tetra­aza­tri­cyclo­[4.4.1.1^3,8^]dodecane [(IV), TATD]. We have previously studied the structure of the 1:1 complex produced by the reaction of TATU with hydro­quinone (Rivera *et al.* 2007 ▸). In that work, we found that the preference for a particular hydrogen-bond inter­action site depends strongly upon the lone-pair orbital hybridization of the N atom. We also demonstrated that a greater degree of *sp* ^3^ character favours the N⋯H—O inter­actions. Later (Rivera *et al.* 2011 ▸), we reported the preparation and structure of an acid–base adduct assembled from TATU and penta­chloro­phenol (PCP). X-ray diffraction analysis of this salt confirmed that the –OH group of the PCP transfers a proton to the N atoms of the aminal moiety. The main consequence of this protonation is the distortion of the cage structure which was attributed to the anomeric effect that governs the aminal group. Studies of phenol complexes with tertiary amines in the solid state show that the proton transfer depends not only on the Δ*K_a_* (p*K_a_* amine − p*K_a_* acid) value, but also on steric and packing effects (Majerz & Sawka-Dobrowolska, 1996 ▸). Because of the acidity of the phenolic group, the proton potential of the hydrogen-bonded system can be fundamentally changed by substituents in the 4-position of the phenol ring (Xiong *et al.*, 2002 ▸). Because of this and as a logical extension of our previous efforts to understand the hydrogen-bonding inter­actions between TATU and phenols, we investigated the reaction of TATU with 4-nitro­phenol. It is worth noting that, contrary to our initial expectation, the substitution of hydro­quinone by 4-nitro­phenol afforded the title compound, (I). Its crystal structure shows a 1:2 stoichiometry. The aminal moiety is linked to two 4-nitro­phenol mol­ecules *via* O—H⋯N hydrogen bonds where one inter­action is to a more basic site (N3) and the other one to an N atom adjacent to the ethyl­ene bridge (N1).
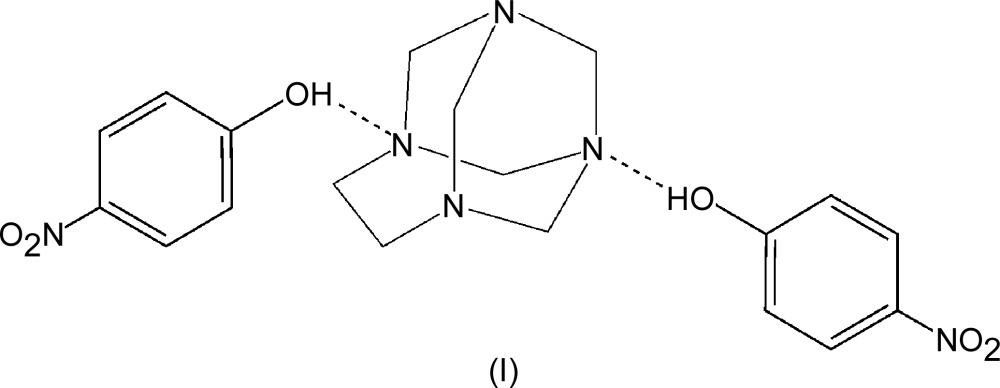


## Structural commentary   

**Figure 1:**
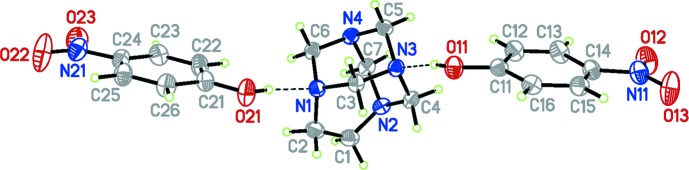
The mol­ecular structure of the title compound, with displacement ellipsoids drawn at the 50% probability level. Hydrogen bonds are drawn as dashed lines.

In the ternary cocrystal of TATU with two mol­ecules of 4-nitro­phenol, (I) (Fig. 1 ▸), the asymmetric unit was chosen such that the two nitro­phenol mol­ecules are linked by two inter­molecular O—H⋯N hydrogen bonds (Table 1 ▸). This arrangement contrasts with related structures (Ng, 2008 ▸; Ng *et al.* 2001 ▸), where the urotropine (HMTA) moiety uses only one of its four N atoms to link to a 4-nitro­phenol mol­ecule *via* a hydrogen bond. It is possible, however, that the presence of a solvent water mol­ecule may influence this observation.

In the three-component aggregates observed here, the O1⋯N3 distance [2.6551 (13) Å], is similar to those observed previously in hydrogen-bond adducts between HMTA and 4-nitro­phenol (Ng, 2008 ▸; Ng *et al.* 2001 ▸). However, this is shorter than the O21⋯N1 distance in the second contact [2.7377 (14) Å]. This is a consequence of the relative proton affinities of the N atoms in TATU (Rivera *et al.*, 2007 ▸). Though this polyamine has four potential protonation sites it is evident that atoms N1 and N3 are not equivalent to one another. In particular, they differ in terms of their pyramidal character which can be estimated from the sum of the bond angles around each N atom, Σα(CNC) (339.5° for N1 and 328.3° for N3). The greater *sp* ^3^ character of the N atom contributes to the increase in proton affinity for N3. The main questions concerning the structure of this ternary cocrystal concerns the site of the formation of the second hydrogen bond. If all N atoms were equivalent, as in HMTA, the second hydrogen-bond inter­action would be possible with any of the amino groups. However, in TATU, as expected, the first hydrogen bond involves more basic site (N3) rather than forming at a site than to adjacent to the ethyl­ene bridge (N1 or N2). For the second hydrogen-bond contact, there are three potential alternative sites. First, a symmetric structure using the more basic N4 site. Alternatively, one of the less basic N1 or N2 atoms linked by the ethyl­ene bridge could be used, resulting in a less symmetric cocrystal.
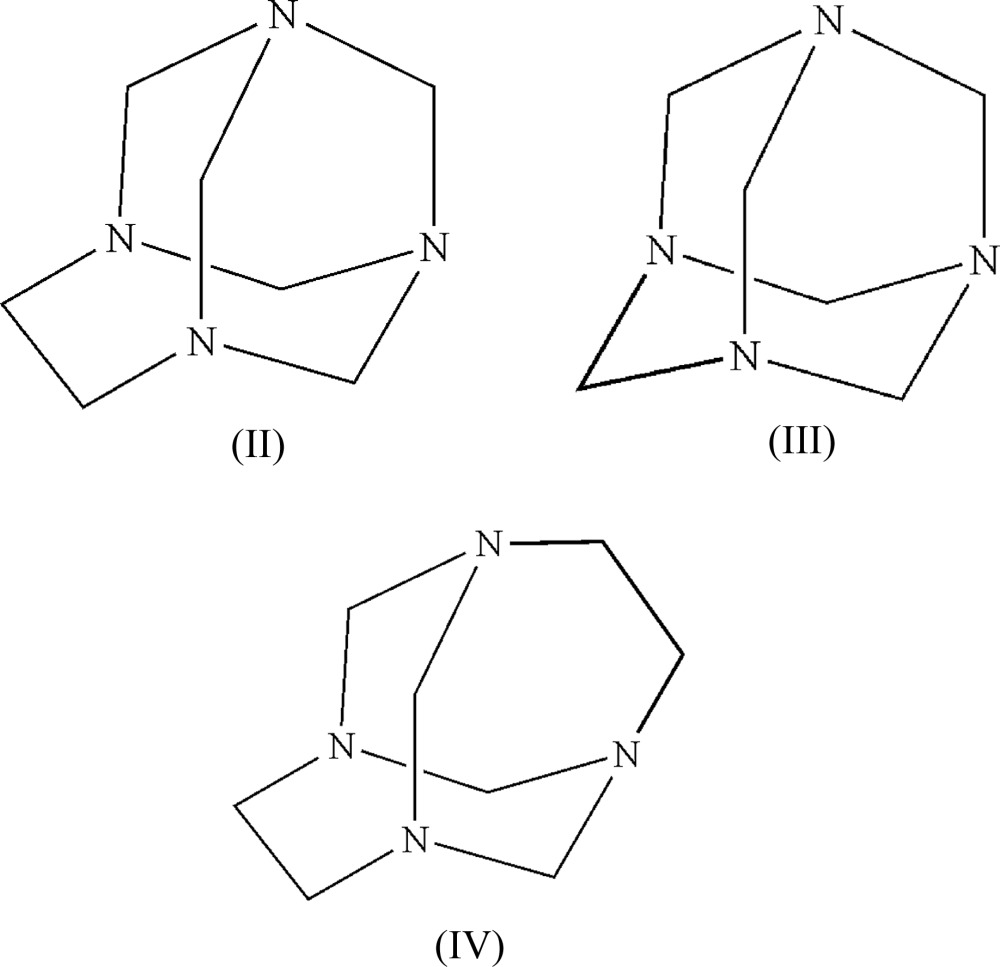


In principle, it might be expected that the supra­molecular structure of the title compound could be considered to be similar to that of the cocrystal formed between hydro­quinone and TATU (Rivera *et al.*, 2007 ▸). In that case, however, the asymmetric unit contains only one half of hydro­quinone mol­ecule and the aminal cage structure, and self-assembly into a symmetric supra­molecular structure via hydrogen bonds join them into a zigzag chain extending along the crystal *b* axis (Rivera *et al.*, 2007 ▸). Certainly each aminal unit links to two hydro­quinone mol­ecules *via* O—H⋯N hydrogen bonds once the symmetry operation is applied, but, in the present case, the asymmetric unit comprises a pair of 4-nitro­phenol mol­ecules and one complete mol­ecule of the aminal cage not related by symmetry elements. Account should also be taken of the effect that the phenol p*K_a_* and the extent of polarization of the N—CH_2_—N bonds upon hydrogen-bond formation. In fact, although the values of the bond lengths and angles in the aminal cage of the title compound are within expected values, there are notable differences when compared to the related TUTU/hydro­quinone system (Rivera *et al.*, 2007 ▸).

Comparison of the C—N bond lengths in the title compound with respect to the mean value of 1.469 Å (Allen *et al.*, 1987 ▸) points out that the presence of strong hydrogen bonds in the title compound affect the length of the CH_2_—N single bonds in the heterocyclic cage system. While in the related structure, the formation of a hydrogen bond with hydro­quinone does not effect the CH_2_—N single-bond lengths significantly [mean values 1.469 (2) Å] (Rivera *et al.*, 2007 ▸), for the title compound, the C5—N3 bond is lengthened [1.4815 (14) Å], while C5—N4 is shortened to 1.4639 (15) Å. In addition, the C4—N3 bond in the title compound is longer than in the related structure by 0.016 Å. The shortest C—N bond within the aminal cage ring in the title compound is the N2—C4 bond at 1.4517 (15) Å. These results are probably connected to presence of the very strong O—H⋯N hydrogen bonds, between the N atoms of the aminal cage structure and the 4-nitro­phenol mol­ecules. Thus, in the title compound, the aminal cage structure acquires a more pronounced anomeric effect due to these hydrogen-bond inter­actions (Alder *et al.*, 1999 ▸). It noteworthy that in the title compound the lengthening of C5—N3 facilitates the inter­action of the nonbonding mol­ecular orbitals of N4 and N2 with the σ*C5—N3 anti­bonding orbital and thus these N atoms are less likely to form the second hydrogen bond and results in the observed second hydrogen-bond inter­action between the N1 atom from the aminal cage and the O21—H21 group of the second 4-nitro­phenol mol­ecule.

The two independent nitro­phenol mol­ecules are essentially planar, with maximum deviations of 0.0157 (13) and 0.0039 (13) Å. The nitro groups are almost coplanar with the aromatic ring plane; the dihedral angles between the planes of the nitro group and the attached benzene rings are 4.04 (17) and 5.79 (17)°. The coplanarity of the nitro groups with the aromatic rings is stabilized by weak C—H⋯O hydrogen bonds between the nitro O atoms and the H atoms of neighbouring structures (Table 1 ▸). In addition, the two of the hy­droxy substituents C—O bonds are similar in length, but are somewhat longer than the normal value for a OH group bound to an aromatic ring (1.362Å; Allen *et al.*, 1987 ▸).

## Supra­molecular features   

**Figure 2:**
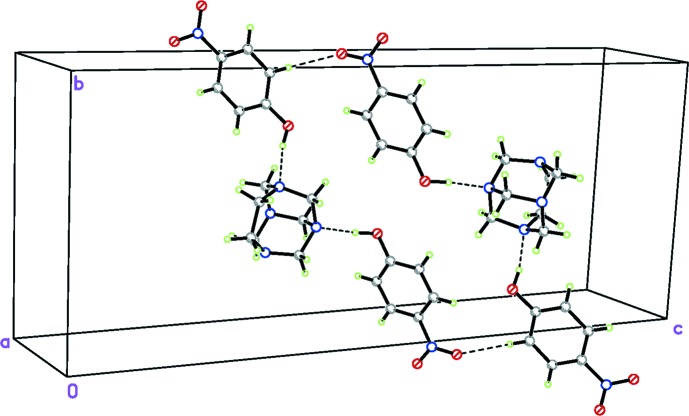
The hydrogen-bonded inversion dimer displaying an 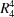
(32) motif in the crystal of the title compound, with hydrogen bonds drawn as dashed lines. [Symmetry code: (i) −*x*, −*y* + 1, −*z* + 1.]

**Figure 3:**
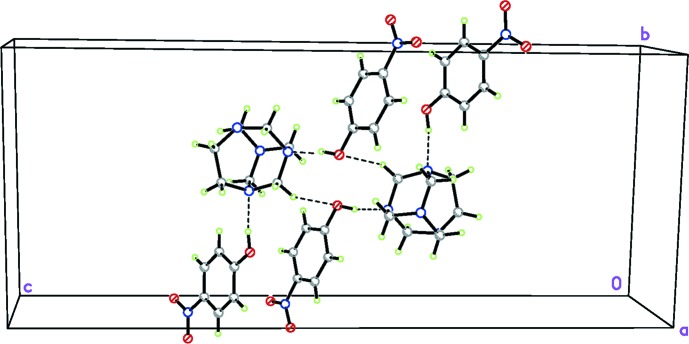
The hydrogen-bonded inversion dimer displaying an 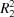
(10) motif in the crystal of the title compound, with hydrogen bonds drawn as dashed lines. [Symmetry code: (ii) −*x* + 1, −*y* + 1, −*z* + 1.]

**Figure 4:**
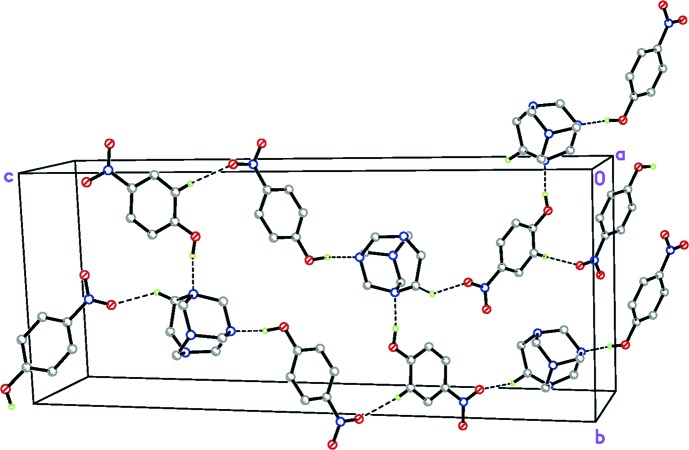
The overall packing of (I). H atoms not involved in hydrogen bonds have been omitted.

In the crystal structure of (I), two 1:2 adducts are linked to one another by C26—H26⋯O13^i^ bond pairs (Table 1 ▸) so that an inversion dimer is formed (Table 1 ▸ and Fig. 2 ▸), which displays an 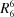
(32) motif (Bernstein *et al.*, 1995 ▸). The hydrogen bond with atom C6 as the donor firms an inversion dimer, generating forming a zigzag chain running parallel to [111] through a second C6—H6*A*⋯O23^iv^ hydrogen bond (Table 1 ▸ and Fig. 4 ▸). Additional C5—H5*A*⋯O11^ii^ inter­molecular hydro­gen-bonding inter­actions form a second supra­molecular inversion dimer with an 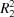
(10) motif (Fig. 3 ▸). Both dimers are further linked by a weak inter­molecular C25—H25⋯N2^v^ hydrogen bond (Table 1 ▸). These contacts combine to generate a three-dimensional network structure.

## Database survey   

Up to now, there are only four structures of 1,3,6,8-tetra­aza­tri­cyclo­[4.3.1.1^3,8^]undecane derivatives in the Cambridge Structural Database (CSD, Version 5.36; Groom & Allen, 2014 ▸), namely hexa­aqua­magnesium dibromide 1,3,6,8-tetra­aza­tri­cyclo­[4.3.1.1^3,8^]undecane tetra­hydrate (Rivera *et al.*, 2014 ▸), 1,3,6,8-tetra­aza­tri­cyclo­[4.3.1.1^3,8^]undecane hydro­quinone (Rivera *et al.* 2007 ▸) and 3,6,8-tri­aza-1-azoniatri­cyclo­[4.3.1.1^3,8^]undecane penta­chloro­phenolate monohydrate (Rivera *et al.*, 2011 ▸). Since 1,3,6,8-tetra­aza­tri­cyclo­[4.3.1.1^3,8^]undecane is a rigid mol­ecule, the torsion angles do not vary considerably even though the C—N bond lengths do.

## Synthesis and crystallization   

Solvent-free direct preparation of the title compound from 1,3,6,8-tetra­aza­tri­cyclo­[4.3.1.1^3,8^]dodecane (TATU) (0.15 g, 1.00 mmol) and 4-nitro­phenol (0.21 g, 1.5 mmol) was carried out by a mechanochemical inter­action in a mortar at room temperature. 30 min were required to complete the reaction. The mixture was then dissolved in a minimum amount of methanol and left to crystallize at room temperature. Subsequent recrystallization with MeOH gave the title compound as colourless crystals in 70% yield (m.p. 388–389 K).

## Refinement   

Crystal data, data collection and structure refinement details are summarized in Table 2 ▸. H atoms were located in a difference map. Those bound to C atoms were positioned geometrically and refined using a riding model with fixed individual displacement parameters [*U*_iso_(H) = 1.2*U*_eq_(C)] and with aromatic C—H = 0.95 Å and methyl­ene C—H = 0.95 Å. H atoms bound to O atoms were refined freely.

## Supplementary Material

Crystal structure: contains datablock(s) I. DOI: 10.1107/S2056989015019659/sj5479sup1.cif

Structure factors: contains datablock(s) I. DOI: 10.1107/S2056989015019659/sj5479Isup2.hkl

Supporting information file. DOI: 10.1107/S2056989015019659/sj5479Isup3.cml

CCDC reference: 1431801

Additional supporting information:  crystallographic information; 3D view; checkCIF report

## Figures and Tables

**Table 1:** Hydrogen-bond geometry (, )

*D*H*A*	*D*H	H*A*	*D* *A*	*D*H*A*
O11H11N3	0.98(2)	1.69(2)	2.6551(13)	167.4(19)
O21H21N1	0.93(2)	1.82(2)	2.7377(14)	168.6(19)
C26H26O13^i^	0.95	2.39	3.3242(17)	166
C5H5*A*O11^ii^	0.99	2.56	3.4940(15)	156
C5H5*B*O12^iii^	0.99	2.53	3.4284(16)	151
C6H6*A*O23^iv^	0.99	2.47	3.4214(16)	160
C25H25N2^v^	0.95	2.60	3.5117(16)	160

Symmetry codes: (i) 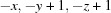; (ii) 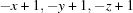; (iii) 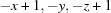; (iv) 
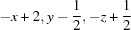
; (v) 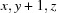.

**Table 2:** Experimental details

Crystal data	
Chemical formula	C_7_H_14_N_4_2C_6_H_5_NO_3_
*M*_r_	432.44
Crystal system, space group	Monoclinic, *P*2_1_/*c*
Temperature (K)	173
*a*, *b*, *c* ()	5.8818(2), 11.9426(4), 28.7820(13)
()	94.252(3)
*V* (^3^)	2016.20(13)
*Z*	4
Radiation type	Mo *K*
(mm^1^)	0.11
Crystal size (mm)	0.29 0.28 0.26

Data collection	
Diffractometer	Stoe IPDS II two-circle
Absorption correction	Multi-scan (*X-AREA*; Stoe Cie, 2001 ▸)
*T*_min_, *T*_max_	0.871, 0.896
No. of measured, independent and observed [*I* > 2(*I*)] reflections	32192, 4379, 4086
*R*_int_	0.066
(sin /)_max_ (^1^)	0.640

Refinement	
*R*[*F* ^2^ > 2(*F* ^2^)], *wR*(*F* ^2^), *S*	0.039, 0.101, 1.05
No. of reflections	4379
No. of parameters	288
H-atom treatment	H atoms treated by a mixture of independent and constrained refinement
_max_, _min_ (e ^3^)	0.28, 0.18

Computer programs: *X-AREA* (Stoe Cie, 2001 ▸), *SHELXS97* and *XP* in *SHELXTL-Plus* (Sheldrick, 2008 ▸) and *SHELXL2014* (Sheldrick, 2015 ▸).

## supplementary crystallographic information

### Crystal data

**Table:**

C_7_H_14_N_4_·2C_6_H_5_NO_3_	*F*(000) = 912
*M**_r_* = 432.44	*D*_x_ = 1.425 Mg m^−^^3^
Monoclinic, *P*2_1_/*c*	Mo *K*α radiation, λ = 0.71073 Å
*a* = 5.8818 (2) Å	Cell parameters from 38374 reflections
*b* = 11.9426 (4) Å	θ = 1.9–27.5°
*c* = 28.7820 (13) Å	µ = 0.11 mm^−^^1^
β = 94.252 (3)°	*T* = 173 K
*V* = 2016.20 (13) Å^3^	Block, colourless
*Z* = 4	0.29 × 0.28 × 0.26 mm

### Data collection

**Table:**

Stoe IPDS II two-circle diffractometer	4086 reflections with *I* > 2σ(*I*)
Radiation source: Genix 3D IµS microfocus X-ray source	*R*_int_ = 0.066
ω scans	θ_max_ = 27.1°, θ_min_ = 1.9°
Absorption correction: multi-scan (*X-AREA*; Stoe & Cie, 2001)	*h* = −7→7
*T*_min_ = 0.871, *T*_max_ = 0.896	*k* = −15→15
32192 measured reflections	*l* = −36→36
4379 independent reflections	

### Refinement

**Table:**

Refinement on *F*^2^	0 restraints
Least-squares matrix: full	Hydrogen site location: mixed
*R*[*F*^2^ > 2σ(*F*^2^)] = 0.039	H atoms treated by a mixture of independent and constrained refinement
*wR*(*F*^2^) = 0.101	*w* = 1/[σ^2^(*F*_o_^2^) + (0.0445*P*)^2^ + 0.6818*P*] where *P* = (*F*_o_^2^ + 2*F*_c_^2^)/3
*S* = 1.05	(Δ/σ)_max_ = 0.001
4379 reflections	Δρ_max_ = 0.28 e Å^−^^3^
288 parameters	Δρ_min_ = −0.18 e Å^−^^3^

### Special details

**Table:**

Geometry. All e.s.d.'s (except the e.s.d. in the dihedral angle between two l.s. planes) are estimated using the full covariance matrix. The cell e.s.d.'s are taken into account individually in the estimation of e.s.d.'s in distances, angles and torsion angles; correlations between e.s.d.'s in cell parameters are only used when they are defined by crystal symmetry. An approximate (isotropic) treatment of cell e.s.d.'s is used for estimating e.s.d.'s involving l.s. planes.

### Fractional atomic coordinates and isotropic or equivalent isotropic displacement parameters (Å^2^)

**Table:**

	*x*	*y*	*z*	*U*_iso_*/*U*_eq_	
N1	0.53235 (16)	0.54216 (8)	0.36281 (3)	0.0257 (2)	
N2	0.53803 (16)	0.30513 (9)	0.34430 (4)	0.0287 (2)	
N3	0.50006 (16)	0.39145 (8)	0.42185 (3)	0.0255 (2)	
N4	0.85374 (16)	0.41212 (8)	0.38454 (3)	0.0259 (2)	
C1	0.4260 (2)	0.37586 (12)	0.30852 (5)	0.0352 (3)	
H1A	0.2678	0.3486	0.3022	0.042*	
H1B	0.5046	0.3656	0.2796	0.042*	
C2	0.4162 (2)	0.50300 (11)	0.31888 (4)	0.0329 (3)	
H2A	0.4823	0.5433	0.2930	0.039*	
H2B	0.2538	0.5251	0.3186	0.039*	
C3	0.42828 (19)	0.50365 (10)	0.40489 (4)	0.0259 (2)	
H3A	0.2606	0.5034	0.3984	0.031*	
H3B	0.4651	0.5585	0.4301	0.031*	
C4	0.43109 (19)	0.30056 (10)	0.38813 (4)	0.0281 (2)	
H4A	0.4678	0.2275	0.4031	0.034*	
H4B	0.2637	0.3038	0.3814	0.034*	
C5	0.75221 (19)	0.39112 (10)	0.42864 (4)	0.0272 (2)	
H5A	0.8027	0.4496	0.4515	0.033*	
H5B	0.8049	0.3177	0.4413	0.033*	
C6	0.78086 (18)	0.52223 (10)	0.36721 (4)	0.0265 (2)	
H6A	0.8402	0.5333	0.3362	0.032*	
H6B	0.8524	0.5797	0.3883	0.032*	
C7	0.78366 (19)	0.32159 (10)	0.35191 (4)	0.0289 (2)	
H7A	0.8454	0.3377	0.3215	0.035*	
H7B	0.8535	0.2508	0.3638	0.035*	
N11	0.0449 (2)	−0.02774 (10)	0.57131 (4)	0.0378 (3)	
O11	0.24196 (16)	0.38395 (7)	0.49365 (3)	0.0338 (2)	
H11	0.351 (3)	0.3797 (17)	0.4697 (7)	0.063 (6)*	
O12	0.1721 (2)	−0.10676 (9)	0.56483 (4)	0.0520 (3)	
O13	−0.1284 (2)	−0.03768 (10)	0.59193 (4)	0.0582 (3)	
C11	0.20153 (19)	0.28382 (10)	0.51277 (4)	0.0270 (2)	
C12	0.3496 (2)	0.19295 (12)	0.50991 (4)	0.0336 (3)	
H12	0.4853	0.2008	0.4942	0.040*	
C13	0.2988 (2)	0.09208 (11)	0.52981 (5)	0.0357 (3)	
H13	0.3992	0.0302	0.5280	0.043*	
C14	0.1002 (2)	0.08132 (10)	0.55258 (4)	0.0299 (3)	
C15	−0.0455 (2)	0.17094 (11)	0.55729 (4)	0.0298 (2)	
H15	−0.1783	0.1630	0.5739	0.036*	
C16	0.0062 (2)	0.27210 (11)	0.53735 (4)	0.0292 (2)	
H16	−0.0920	0.3345	0.5404	0.035*	
N21	0.9793 (2)	1.06407 (10)	0.27085 (4)	0.0391 (3)	
O21	0.44401 (17)	0.76721 (8)	0.36343 (4)	0.0410 (2)	
H21	0.490 (3)	0.6929 (19)	0.3610 (7)	0.065 (6)*	
O22	0.9280 (3)	1.16343 (9)	0.27052 (5)	0.0697 (4)	
O23	1.13483 (19)	1.02708 (10)	0.24912 (4)	0.0512 (3)	
C21	0.5798 (2)	0.83634 (10)	0.34109 (4)	0.0313 (3)	
C22	0.7709 (2)	0.79931 (11)	0.31941 (5)	0.0358 (3)	
H22	0.8091	0.7220	0.3200	0.043*	
C23	0.9043 (2)	0.87407 (11)	0.29716 (4)	0.0346 (3)	
H23	1.0354	0.8491	0.2828	0.042*	
C24	0.8445 (2)	0.98616 (10)	0.29611 (4)	0.0306 (3)	
C25	0.6549 (2)	1.02526 (10)	0.31720 (5)	0.0346 (3)	
H25	0.6164	1.1025	0.3161	0.042*	
C26	0.5237 (2)	0.94998 (11)	0.33973 (5)	0.0350 (3)	
H26	0.3941	0.9756	0.3545	0.042*	

### Atomic displacement parameters (Å^2^)

**Table:**

	*U*^11^	*U*^22^	*U*^33^	*U*^12^	*U*^13^	*U*^23^
N1	0.0219 (4)	0.0270 (5)	0.0285 (5)	0.0018 (4)	0.0044 (4)	0.0031 (4)
N2	0.0257 (5)	0.0298 (5)	0.0305 (5)	−0.0023 (4)	0.0029 (4)	−0.0042 (4)
N3	0.0242 (5)	0.0247 (5)	0.0280 (5)	0.0003 (4)	0.0043 (4)	0.0014 (4)
N4	0.0209 (4)	0.0249 (5)	0.0321 (5)	0.0007 (4)	0.0025 (4)	−0.0009 (4)
C1	0.0324 (6)	0.0422 (7)	0.0305 (6)	−0.0025 (5)	−0.0016 (5)	−0.0024 (5)
C2	0.0281 (6)	0.0403 (7)	0.0299 (6)	0.0022 (5)	0.0000 (5)	0.0045 (5)
C3	0.0238 (5)	0.0253 (5)	0.0294 (6)	0.0028 (4)	0.0069 (4)	0.0010 (4)
C4	0.0243 (5)	0.0257 (5)	0.0345 (6)	−0.0033 (4)	0.0040 (4)	−0.0004 (4)
C5	0.0248 (5)	0.0277 (6)	0.0287 (5)	0.0015 (4)	−0.0006 (4)	0.0007 (4)
C6	0.0220 (5)	0.0253 (5)	0.0326 (6)	−0.0011 (4)	0.0052 (4)	0.0006 (4)
C7	0.0247 (5)	0.0277 (6)	0.0349 (6)	0.0016 (4)	0.0064 (4)	−0.0043 (5)
N11	0.0488 (7)	0.0338 (6)	0.0318 (5)	0.0017 (5)	0.0095 (5)	0.0019 (4)
O11	0.0399 (5)	0.0307 (5)	0.0321 (4)	0.0000 (4)	0.0112 (4)	0.0006 (3)
O12	0.0651 (7)	0.0342 (5)	0.0579 (7)	0.0130 (5)	0.0134 (5)	0.0087 (5)
O13	0.0711 (8)	0.0441 (6)	0.0646 (7)	−0.0059 (6)	0.0398 (6)	0.0042 (5)
C11	0.0284 (5)	0.0311 (6)	0.0216 (5)	−0.0005 (4)	0.0025 (4)	−0.0016 (4)
C12	0.0281 (6)	0.0415 (7)	0.0326 (6)	0.0059 (5)	0.0112 (5)	0.0033 (5)
C13	0.0368 (7)	0.0374 (7)	0.0342 (6)	0.0124 (5)	0.0121 (5)	0.0035 (5)
C14	0.0348 (6)	0.0311 (6)	0.0244 (5)	0.0023 (5)	0.0055 (4)	0.0007 (4)
C15	0.0272 (5)	0.0367 (6)	0.0264 (5)	0.0011 (5)	0.0072 (4)	−0.0018 (5)
C16	0.0285 (5)	0.0326 (6)	0.0268 (5)	0.0058 (5)	0.0053 (4)	−0.0029 (4)
N21	0.0504 (7)	0.0367 (6)	0.0316 (5)	−0.0106 (5)	0.0114 (5)	−0.0037 (4)
O21	0.0439 (5)	0.0282 (5)	0.0538 (6)	0.0033 (4)	0.0227 (4)	0.0059 (4)
O22	0.1072 (11)	0.0299 (6)	0.0785 (9)	−0.0138 (6)	0.0509 (8)	−0.0044 (5)
O23	0.0515 (6)	0.0554 (7)	0.0498 (6)	−0.0025 (5)	0.0240 (5)	0.0062 (5)
C21	0.0353 (6)	0.0284 (6)	0.0309 (6)	0.0009 (5)	0.0070 (5)	0.0021 (5)
C22	0.0416 (7)	0.0280 (6)	0.0391 (7)	0.0068 (5)	0.0120 (5)	0.0042 (5)
C23	0.0367 (6)	0.0357 (7)	0.0326 (6)	0.0038 (5)	0.0102 (5)	0.0006 (5)
C24	0.0376 (6)	0.0301 (6)	0.0247 (5)	−0.0060 (5)	0.0056 (5)	−0.0021 (4)
C25	0.0449 (7)	0.0241 (6)	0.0356 (6)	0.0000 (5)	0.0082 (5)	−0.0034 (5)
C26	0.0375 (6)	0.0297 (6)	0.0392 (7)	0.0034 (5)	0.0126 (5)	−0.0023 (5)

### Geometric parameters (Å, º)

**Table:**

N1—C2	1.4685 (16)	N11—C14	1.4557 (16)
N1—C3	1.4707 (14)	O11—C11	1.3449 (15)
N1—C6	1.4771 (14)	O11—H11	0.98 (2)
N2—C1	1.4517 (16)	C11—C12	1.3977 (17)
N2—C4	1.4517 (15)	C11—C16	1.4005 (16)
N2—C7	1.4584 (15)	C12—C13	1.3762 (19)
N3—C3	1.4770 (14)	C12—H12	0.9500
N3—C5	1.4815 (14)	C13—C14	1.3872 (17)
N3—C4	1.4920 (15)	C13—H13	0.9500
N4—C6	1.4595 (15)	C14—C15	1.3839 (17)
N4—C5	1.4639 (15)	C15—C16	1.3809 (18)
N4—C7	1.4708 (15)	C15—H15	0.9500
C1—C2	1.5493 (19)	C16—H16	0.9500
C1—H1A	0.9900	N21—O22	1.2242 (17)
C1—H1B	0.9900	N21—O23	1.2280 (16)
C2—H2A	0.9900	N21—C24	1.4518 (16)
C2—H2B	0.9900	O21—C21	1.3451 (15)
C3—H3A	0.9900	O21—H21	0.93 (2)
C3—H3B	0.9900	C21—C26	1.3965 (18)
C4—H4A	0.9900	C21—C22	1.3972 (18)
C4—H4B	0.9900	C22—C23	1.3776 (18)
C5—H5A	0.9900	C22—H22	0.9500
C5—H5B	0.9900	C23—C24	1.3838 (18)
C6—H6A	0.9900	C23—H23	0.9500
C6—H6B	0.9900	C24—C25	1.3899 (18)
C7—H7A	0.9900	C25—C26	1.3777 (18)
C7—H7B	0.9900	C25—H25	0.9500
N11—O13	1.2225 (16)	C26—H26	0.9500
N11—O12	1.2272 (16)		
			
C2—N1—C3	114.45 (9)	N2—C7—H7A	108.5
C2—N1—C6	114.63 (9)	N4—C7—H7A	108.5
C3—N1—C6	110.41 (9)	N2—C7—H7B	108.5
C1—N2—C4	115.79 (10)	N4—C7—H7B	108.5
C1—N2—C7	114.87 (10)	H7A—C7—H7B	107.5
C4—N2—C7	111.27 (9)	O13—N11—O12	122.61 (12)
C3—N3—C5	107.75 (9)	O13—N11—C14	118.94 (11)
C3—N3—C4	112.82 (9)	O12—N11—C14	118.43 (11)
C5—N3—C4	107.77 (9)	C11—O11—H11	112.8 (12)
C6—N4—C5	108.81 (9)	O11—C11—C12	122.44 (11)
C6—N4—C7	112.51 (9)	O11—C11—C16	118.17 (11)
C5—N4—C7	108.34 (9)	C12—C11—C16	119.37 (11)
N2—C1—C2	117.08 (10)	C13—C12—C11	119.97 (11)
N2—C1—H1A	108.0	C13—C12—H12	120.0
C2—C1—H1A	108.0	C11—C12—H12	120.0
N2—C1—H1B	108.0	C12—C13—C14	119.62 (12)
C2—C1—H1B	108.0	C12—C13—H13	120.2
H1A—C1—H1B	107.3	C14—C13—H13	120.2
N1—C2—C1	117.14 (10)	C15—C14—C13	121.59 (12)
N1—C2—H2A	108.0	C15—C14—N11	119.91 (11)
C1—C2—H2A	108.0	C13—C14—N11	118.49 (11)
N1—C2—H2B	108.0	C16—C15—C14	118.63 (11)
C1—C2—H2B	108.0	C16—C15—H15	120.7
H2A—C2—H2B	107.3	C14—C15—H15	120.7
N1—C3—N3	115.44 (9)	C15—C16—C11	120.75 (11)
N1—C3—H3A	108.4	C15—C16—H16	119.6
N3—C3—H3A	108.4	C11—C16—H16	119.6
N1—C3—H3B	108.4	O22—N21—O23	122.49 (12)
N3—C3—H3B	108.4	O22—N21—C24	118.80 (12)
H3A—C3—H3B	107.5	O23—N21—C24	118.64 (12)
N2—C4—N3	115.05 (9)	C21—O21—H21	111.1 (13)
N2—C4—H4A	108.5	O21—C21—C26	117.55 (11)
N3—C4—H4A	108.5	O21—C21—C22	123.03 (11)
N2—C4—H4B	108.5	C26—C21—C22	119.42 (12)
N3—C4—H4B	108.5	C23—C22—C21	120.49 (12)
H4A—C4—H4B	107.5	C23—C22—H22	119.8
N4—C5—N3	110.72 (9)	C21—C22—H22	119.8
N4—C5—H5A	109.5	C22—C23—C24	118.98 (12)
N3—C5—H5A	109.5	C22—C23—H23	120.5
N4—C5—H5B	109.5	C24—C23—H23	120.5
N3—C5—H5B	109.5	C23—C24—C25	121.76 (11)
H5A—C5—H5B	108.1	C23—C24—N21	118.93 (11)
N4—C6—N1	115.99 (9)	C25—C24—N21	119.28 (11)
N4—C6—H6A	108.3	C26—C25—C24	118.81 (12)
N1—C6—H6A	108.3	C26—C25—H25	120.6
N4—C6—H6B	108.3	C24—C25—H25	120.6
N1—C6—H6B	108.3	C25—C26—C21	120.54 (12)
H6A—C6—H6B	107.4	C25—C26—H26	119.7
N2—C7—N4	115.00 (9)	C21—C26—H26	119.7
			
C4—N2—C1—C2	63.86 (14)	C11—C12—C13—C14	0.1 (2)
C7—N2—C1—C2	−68.06 (14)	C12—C13—C14—C15	−2.3 (2)
C3—N1—C2—C1	−67.26 (13)	C12—C13—C14—N11	177.01 (12)
C6—N1—C2—C1	61.78 (14)	O13—N11—C14—C15	−1.58 (19)
N2—C1—C2—N1	3.43 (16)	O12—N11—C14—C15	176.86 (12)
C2—N1—C3—N3	84.49 (12)	O13—N11—C14—C13	179.10 (13)
C6—N1—C3—N3	−46.64 (13)	O12—N11—C14—C13	−2.46 (18)
C5—N3—C3—N1	54.85 (12)	C13—C14—C15—C16	2.17 (19)
C4—N3—C3—N1	−63.99 (12)	N11—C14—C15—C16	−177.13 (11)
C1—N2—C4—N3	−85.86 (12)	C14—C15—C16—C11	0.16 (18)
C7—N2—C4—N3	47.73 (13)	O11—C11—C16—C15	179.27 (11)
C3—N3—C4—N2	64.93 (12)	C12—C11—C16—C15	−2.31 (18)
C5—N3—C4—N2	−53.89 (12)	O21—C21—C22—C23	−179.78 (13)
C6—N4—C5—N3	61.14 (11)	C26—C21—C22—C23	0.5 (2)
C7—N4—C5—N3	−61.47 (11)	C21—C22—C23—C24	−0.8 (2)
C3—N3—C5—N4	−61.52 (11)	C22—C23—C24—C25	0.5 (2)
C4—N3—C5—N4	60.50 (11)	C22—C23—C24—N21	−177.24 (12)
C5—N4—C6—N1	−53.68 (12)	O22—N21—C24—C23	−178.98 (14)
C7—N4—C6—N1	66.39 (12)	O23—N21—C24—C23	3.85 (19)
C2—N1—C6—N4	−85.14 (12)	O22—N21—C24—C25	3.2 (2)
C3—N1—C6—N4	45.89 (13)	O23—N21—C24—C25	−173.99 (13)
C1—N2—C7—N4	85.71 (13)	C23—C24—C25—C26	0.0 (2)
C4—N2—C7—N4	−48.33 (13)	N21—C24—C25—C26	177.83 (12)
C6—N4—C7—N2	−64.98 (13)	C24—C25—C26—C21	−0.4 (2)
C5—N4—C7—N2	55.35 (13)	O21—C21—C26—C25	−179.64 (12)
O11—C11—C12—C13	−179.47 (12)	C22—C21—C26—C25	0.1 (2)
C16—C11—C12—C13	2.18 (19)		

### Hydrogen-bond geometry (Å, º)

**Table:**

*D*—H···*A*	*D*—H	H···*A*	*D*···*A*	*D*—H···*A*
O11—H11···N3	0.98 (2)	1.69 (2)	2.6551 (13)	167.4 (19)
O21—H21···N1	0.93 (2)	1.82 (2)	2.7377 (14)	168.6 (19)
C26—H26···O13^i^	0.95	2.39	3.3242 (17)	166
C5—H5*A*···O11^ii^	0.99	2.56	3.4940 (15)	156
C5—H5*B*···O12^iii^	0.99	2.53	3.4284 (16)	151
C6—H6*A*···O23^iv^	0.99	2.47	3.4214 (16)	160
C25—H25···N2^v^	0.95	2.60	3.5117 (16)	160

Symmetry codes: (i) −*x*, −*y*+1, −*z*+1; (ii) −*x*+1, −*y*+1, −*z*+1; (iii) −*x*+1, −*y*, −*z*+1; (iv) −*x*+2, *y*−1/2, −*z*+1/2; (v) *x*, *y*+1, *z*.
